# Use of Tongue Blade to Reposition Palatally Luxated Tooth due to Trauma: A Novel Technique

**DOI:** 10.5005/jp-journals-10005-1167

**Published:** 2012-12-05

**Authors:** Akhilesh Sharma, Amitha M Hegde

**Affiliations:** Senior Lecturer, Department of Pedodontics and Preventive Children Dentistry, Dayananda Sagar College of Dental Sciences, Bengaluru Karnataka, India, e-mail: akhilhere@yahoo.com; Professor and Head, Department of Pedodontics and Preventive Children Dentistry, AB Shetty Memorial Institute of Dental Sciences, Mangalore Karnataka, India

**Keywords:** Luxation, Tongue blade, Repositioning teeth, Trauma

## Abstract

Luxational injuries to the permanent anterior teeth in children are a cause of concern. Palatal luxation of maxillary left central incisor with bleeding of gingival sulcus and fracture of maxillary right central incisor involving enamel and dentine in a 9-year- old girl is presented. The dental occlusion was deranged due to the luxation. Management consisted of repositioning of the luxated tooth using tongue blade under local anesthesia and composite build up of the fractured incisor. Tooth was stable in position with intact occlusion and no loss of vitality of pulp with a follow-up of 2 years. Tongue blade can be used as an alternative to forceful manual repositioning of teeth in selected cases.

**How to cite this article:** Sharma A, Hegde AM. Use of Tongue Blade to Reposition Palatally Luxated Tooth due to Trauma: A Novel Technique. Int J Clin Pediatr Dent 2012;5(3):207-208.

## INTRODUCTION

Dental injuries are considered emergency situation that require immediate care.^1^ In addition to the physical trauma, emotionally these injuries involve not only the child but also the accompanying person, thus representing a challenge to the dentist.^2^

Luxation injuries comprise 15 to 61% of dental traumas to permanent teeth with a peak in the age group of 8 to 12 years. Predominant etiologic factors of luxation injuries in permanent dentition are bicycle injuries, falls, fights and sports injuries.^3^ Frequently, two or more teeth are luxated simultaneously and a number of luxations show concomitant crown or root fractures. Laterally luxated teeth usually have their crowns displaced lingually and are often associated with fractures of the vestibular part of the socket wall. Displacement of teeth after lateral luxation is normally evident by visual inspection. However, in case of marked inclination of maxillary teeth, it can be difficult to decide whether the trauma has caused minor abnormalities in tooth position. In such cases, occlusion should be checked.^3^

## CASE REPORT

**Fig. 1:**
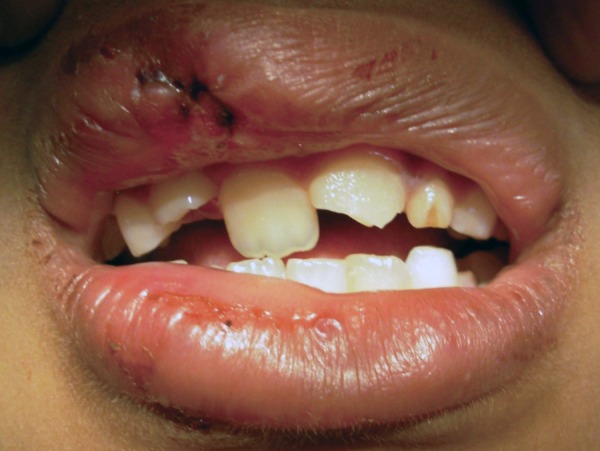
Preoperative–palatal luxation of 11 and Ellis class II fracture of 21 and lip swelling

This is a case of 9-year-old girl who reported to the department of pedodontics and preventive dentistry with a history of trauma to the teeth due to a fall 2 hours ago. Patient had a fall during play. On extraoral examination patient had straight profile and swelling of upper and lower lip on right side with upper lip laceration. Intraorally permanent maxillary left central incisor had class II Ellis fracture and permanent maxillary right central incisor was luxated palatally (Fig. 1) with bleeding of the gingiva. Patient had mixed dentition and occlusion was deranged. Permanent maxillary right central incisor was firm and had metallic sound on percussion. There was no associated alveolar bone fracture. Periapical radiograph of permanent maxillary left central incisor confirmed class II Ellis fracture.

**Fig. 2:**
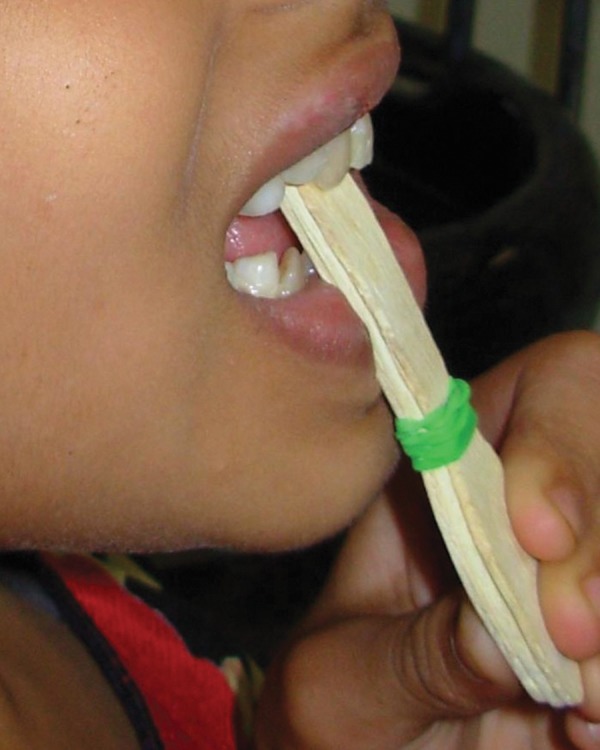
Placement of tongue blade

**Fig. 3:**
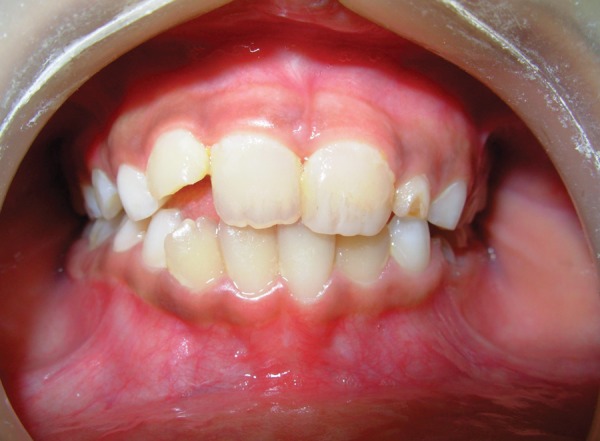
Postoperative–correction of luxation of 11 and composite build up of 21 with establishment of occlusion

Treatment consisted of cleaning the lips and the oral cavity with saline. Composite build up of permanent maxillary left central incisor was done. Regarding the luxated tooth permanent maxillary right central incisor, we have used a novel approach of using a tongue blade to reposition the tooth. Local anesthesia; infraorbital nerve block was administered and the child was shown how to hold the tongue blade and apply a constant pressure on the tooth permanent maxillary right central incisor (Fig. 2). After 10 minutes the occlusion was checked and was found to be corrected (Fig. 3). The child was given instructions not to open the bite until next 30 minutes and was advised to do the tongue blade holding activity just once more at home. Follow-up after a week demonstrated the corrected position of the tooth. The edema had subsided, laceration of lip healed and the gingiva on the palatal side of permanent maxillary right central incisor appeared normal.

## DISCUSSION

The goal of treatment for traumatically injured teeth is to return the teeth to acceptable function and appearance. Normal function (if present before the traumatic event) requires repositioning of the teeth if they were displaced, and acceptable appearance requires repair of possible dental fractures and proper positioning of peridental soft tissues.^4^

Laterally, luxated teeth often have their crowns displaced palatally^5^ and can easily be diagnosed clinically by visual inspection. Treatment of laterally luxated teeth is repositioning of the teeth immediately. Repositioning is a forceful procedure and traumatogenic.^6^ Repositioning can be done manually or with the forceps. It involves extrusion of the tooth from socket and its repositioning followed by compression of the labial and palatal bone with digital pressure to facilitate periodontal healing.^7^ The palatal luxation reported in the present case was minor and the patient reported immediately within 2 hours of trauma. Tongue blade has been used as a conservative method of correcting developing anterior crossbite. Continuous application of pressure from tongue blade has shown to correct the crossbite in 30 minutes without any discomfort to the patient. The conventional method of repositioning would be forceful and traumatic, thus tongue blade was used to apply force to reposition the tooth and to free it from lingual locking. The child applied continuous force at 45° to the tooth luxated. After 10 minutes the tooth position was corrected. The child was asked not to release bite for 30 minutes and advised soft diet. The advantage of using the current method was that it is a traumatic, not forceful, takes less clinical time, low cost and causes minimal psychological trauma to the child. The response of the tooth to the correcting pressure of the tongue blade was rapid.^8^ Crown fractures involving dentin leads to ingress of saliva and bacteria in to the dentinal tubules. The treatment of choice involves placing a protective base and an acid-etch composite restoration.^9^ Further recall at 1 week the occlusion intact and patient was asymptomatic. Pulp testing at 1, 3, 6 months and 2 years recall was positive.

## CONCLUSION

Luxational injuries require immediate repositioning in order to maintain the correct tooth position and cause minimal or no trauma to the pulpal vitality of the tooth. Atraumatic, simple, less time consuming method of using a tongue blade can be successfully used to reposition a palatally luxated tooth.
